# Effectiveness of a multimodal pain management concept for patients with cervical radiculopathy with focus on cervical epidural injections

**DOI:** 10.1038/s41598-017-08350-x

**Published:** 2017-08-11

**Authors:** Achim Benditz, Melanie Brunner, Florian Zeman, Felix Greimel, Völlner Florian, Daniel Boluki, Joachim Grifka, Markus Weber, Tobias Renkawitz

**Affiliations:** 1 Department of Orthopaedics, University Medical Centre Regensburg, Asklepios Klinikum Bad Abbach, Kaiser-Karl-V-Allee 3, 93077 Bad Abbach, Germany; 2 Centre for Clinical Studies, University Medical Centre Regensburg, Franz-Josef-Strauss-Allee 11, 93053 Regensburg, Germany

## Abstract

Cervical radiculopathy has become an increasing problem worldwide. Conservative treatment options have been recommended in many reviews on cervical radiculopathy, ranging from different types of physiotherapy to waiting for remission by natural history. No multimodal pain management concept (MPM) on an inpatient basis has been evaluated. This study aimed at showing the positive short-term effects of an inpatient multimodal pain management concept with focus on cervical translaminar epidural steroid injection for patients with cervical radiculopathy. 54 patients who had undergone inpatient MPM for 10 days were evaluated before and after 10-days treatment. The NRS (0–10) value for arm pain could be reduced from 6.0 (IQR 5.7–6.8) to 2.25 (IQR 2.0–3.1) and from 5.9 (IQR 4.8–6.0) to 2.0 (IQR 1.7–2.6) for neck pain. Neck pain was reduced by 57.4% and arm pain by 62.5%. 2 days after epidural steroid injection, pain was reduced by 40.1% in the neck and by 43.4% in the arms. MPM seems to be an efficient short-term approach to treating cervical radiculopathy. Cervical translaminar epidural steroid injection is an important part of this concept. In the absence of a clear indication for surgery, MPM represents a treatment option.

## Introduction

The incidence of neck pain in clinical studies ranges between 10.4% and 71.5%, and the annual prevalence is estimated to vary between 30% and 50%^1–6^. 2009; Hoy 2010] The average annual age-adjusted incidence rates per 100,000 population for cervical radiculopathy are 83.2 and age-specific 202.9 in the age group 50–54 years^7^. Cervical radiculopathy has become an increasing problem over the last years. E.g. in Germany, the number of patients with cervical radiculopathy and inpatient treatment doubled from 2005 to 2015^8^.

The degenerative changes mainly affect the lower levels of the cervical spine, in particular C5/C6 as well as C6/C7 and rare C7/Th1 or C4/C5. In our clinical experience, radiologically elevated findings in native and sectional image diagnosis (CT, MRI) correlate only partially with clinical findings. Cervical radiculopathy is resulting from nerve root dysfunction, which may be caused by several reasons, most commonly by disc herniation and spinal foraminal stenosis^9^. In addition, there may be other reasons for upper extremity pain than radiculopathy. Muscles, ligaments or cervical facet joints may be responsible for pain in the neck or in the upper extremities^1, 3, 10, 11^. ‘Clinically, it is characterized by arm pain, in some cases paraesthesia and eventually reduced muscle strength, altered sensation and impairment of deep tendon reflexes’^12, 13^.

The age group with the highest risk of developing cervical radiculopathy in the USA and European countries is that of people aged between 35 and 49 years^14^.

Most authors have viewed pain episodes over a person’s lifetime as common relapses. Women and residents of high-income countries or urban areas have a higher risk of developing cervical radiculopathy^14^.

Furthermore, neck pain has a significant impact on socioeconomic factors and on costs of public health services^15–17^. A report describing the U.S. health status regarding diseases and risk factors, neck pain ranked number 4 between 1990 and 2010^1, 15^.

In addition, Nolet *et al*. proposed that neck pain contributes to poor physical quality of life in the future^17^. The costs of public health services are steadily increasing, while the number of effective treatment options is still limited; therefore, it is very important to find the right treatment for each individual patient as soon as possible^18^.

The number of surgical interventions for cervical radiculopathy and degenerative conditions have grown rapidly in the United States^19, 20^.

This increase may be due to the low reimbursement of conservative treatments by health care providers and the lack of sufficient evidence for conservative treatment options for neck and arm pain. In Germany for example, the inclusion criteria for an inpatient multimodal pain management concept (MPM) are so tight, that in our daily clinical experience only 20% of patients can benefit from it. The therapeutic concepts available are very heterogeneous and can hardly be compared. In our opinion, in the absence of a clear indication for surgery, intensive conservative treatment should be provided, and all conservative treatment options should be exhausted prior to any spine surgery^21^. The mostly non-surgical intervention described in the literature so far is epidural steroid injection, which is still listed as ‘off-label’ use^22^. Nevertheless, literature reports have often described the success of this treatment method for pain relief from radicular symptoms^1, 3, 10, 11, 23–31^. Injection techniques have become an important tool for treating spinal pain^24^.

In the case of low back pain, injection therapy is often accompanied by multimodal treatment such as physical therapy and psychological counselling to avoid pain chronification^32^. MPM has been shown to be a very beneficial treatment option for avoiding surgery and reducing pain in patients with radicular nerve root compression^33–35^. Beside the injections, conservative treatment options have been recommended in many reviews on cervical radiculopathy, ranging from different types of physiotherapy to waiting for remission by natural history. There are studies to show the efficacy of training programs on upper extremity pain in patients with chronic neck in the short and long term^36–38^.

However, no multimodal treatment concept on an inpatient basis has yet been evaluated^9, 39, 40^. Only little is known about the use of multimodal pain management concepts in the case of cervical radiculopathy. Therefore, it is important to show the effectiveness of a multimodal pain management concept and particularly the benefits of epidural steroid injection within this concept. Due to the nerve root injections, pain and symptoms can be reduced faster and the patient can benefit from the exercise programs and the behavioural approach within the hospital stay. The multimodal approach including orthopaedic and psychological interventions helps to stop the vicious circle.

## Aim of the study

This study aimed at showing the short-term effect of an inpatient multimodal therapeutic concept based on drug injections – with particular focus on cervical translaminar epidural steroid injection – for patients with cervical radiculopathy.

## Methods

This non-randomized, unblended, prospective, clinical study included male and female patients with cervical radiculopathy, who had been treated according to a multimodal therapeutic concept at the Department of Orthopaedics of the University Medical Centre Regensburg between March 2015 and September 2015. Participation in the study was voluntary. Inclusion criteria were neck and arm pain >4 on a numeric rating scale (NRS 0–10) and clear signs of radiculopathy, which means radiating arm pain, positive Spurling test and according MRI findings^41^. In addition, to be included in the follow-up, patients had to have participated in at least two psychological sessions during therapy. An absolute indication for surgery had to be excluded and a clear indication for MPM was required. That means at least 6 weeks of pain and failed outpatient treatment. Exclusion criteria were cervical surgery before treatment, myelopathy, tumours with spinal involvement, and congenital spinal deformities. In addition, patients had to speak German language to be able to take part in the psychological behavioral education lessons. Since this was a single-arm study, no control group was available. Cervical nerve root injections may have some severe side effects; therefore, placebo injections cannot be justified^42^. The study was approved by the Ethics Commission of the University of Regensburg (24 February 2015, reference no. 16-101-0014) and carried out in accordance to the approved guidelines of the Helsinki Declaration of 1975. A written informed consent was obtained from all study participants. The study is registered on 22.02.2017 in the German Clinical Trials Register (Deutsches Register Klinischer Studien; DRKS) under the number DRKS00011788 (WHO register).

### Patients

**Figure 1:**
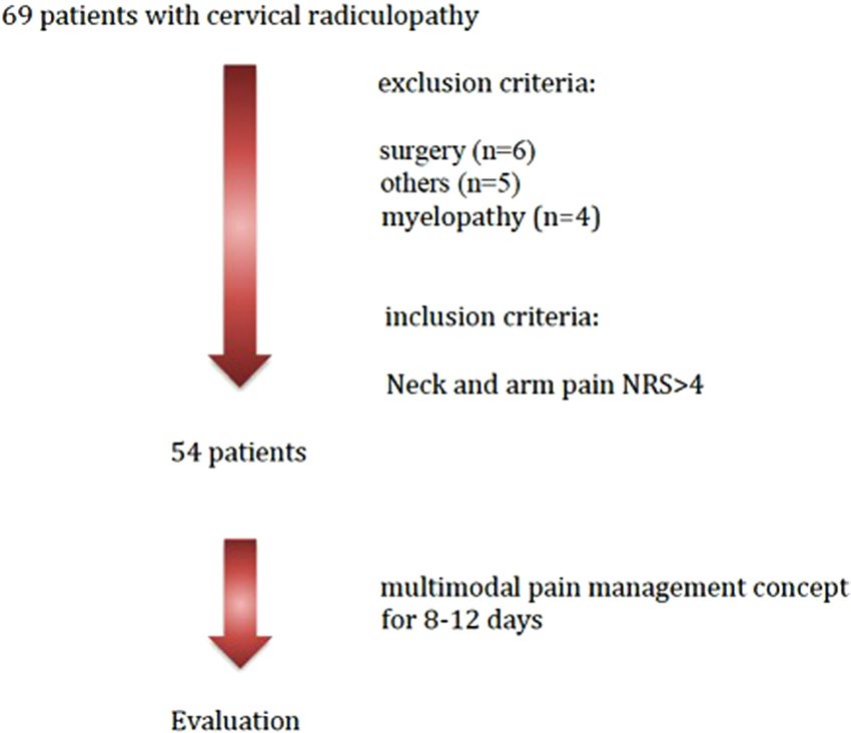
Flowchart of patient inclusion.

The patients were recruited for MPM treatment in our outpatient clinic when fulfilling the described criteria. The inclusion in the study was done on their first day of hospital stay by the first author. 54 of initially 69 patients remained after evaluating the exclusion criteria. 6 of the 11 excluded patients had received primary surgery, 4 showed myelopathy in the MRI, and 5 declined participation. (Fig. 1) The average age of excluded patients is 52 years (32–63), 7 female and 4 male patients.

In all patients, diagnosis of cervical radiculopathy was made by means of patient history and clinical symptoms prior to treatment. All 54 patients also showed relevant findings in the MRI (disc herniation or recess stenosis), so that injection-based therapy represented an appropriate treatment option. The age of the included patients was between 29 and 79 years. The relevant demographic data can be seen in Table 1. Only 6 patients (11.1%) had experienced neck and arm pain for just 6 weeks, 19 (35.2%) for more than 3 months and 18 (33.3%) for more than 2 years. The remaining 11 (20.4) patients had experienced pain between 6 and 12 weeks before treatment. All patients had neck and arm pain, 52.6% of them also pseudo-radicular pain and 47.4% clear radicular pain.

**Table 1:** Demographic data of the patient group (mean and range).

	Women (n = 32)	Men (n = 22)	Together (n = 54)
**Age (years)**	58 (31–87) SD 15.1	55 (33–66) SD 8.3	57 (31–87) SD 12.8
**BMI**	27.9 (19.5–43.2)	28.9 (19.5–39.8)	28.32 (19.5–43.2)
**HADS anxiety**	normal 9 (28.1%) borderline 12 (37.5%) abnormal 11 (34.4%)	normal 9 (40.9%) borderline 7 (31.8%) abnormal 6 (27.3%)	normal 18 (33.3%) borderline 19 (35.2%) abnormal (31.5%)
**HADS depression**	normal 19 (59.4%) borderline 6 (18.8%) abnormal 7 (21.9%)	normal 14 (63.3%) borderline 4 (18.2%) abnormal 4 (18.2%)	normal 33 (61.1%) borderline 10 (18.5%) abnormal 11 (20.4%)
**affected nerve root**	C5: 13 (40.6%) C6: 15 (46.9%) C7: 4 (12.5%)	C5: 8 (36.4%) C6: 10 (45.5%) C7: 4 (18.2%)	C5: 21 (38.9%) C6: 25 (46.3%) C7: 8 (14.8%)
**Work status when starting treatment**	working 15 (46.9%) ill reported 5 (15.6%) retired 12 (37.5%)	working 13 (59.1%) ill reported 7 (31.8%) retired 2 (9.1%)	working 28 (51.9%) ill reported 12 (22.2%) retired 14 (25.9%)

### Intervention

This multimodal pain management concept has already been published several times by the author for low back pain^32^ and has now been adapted for patients with cervical radiculopathy.

**Figure 2:**
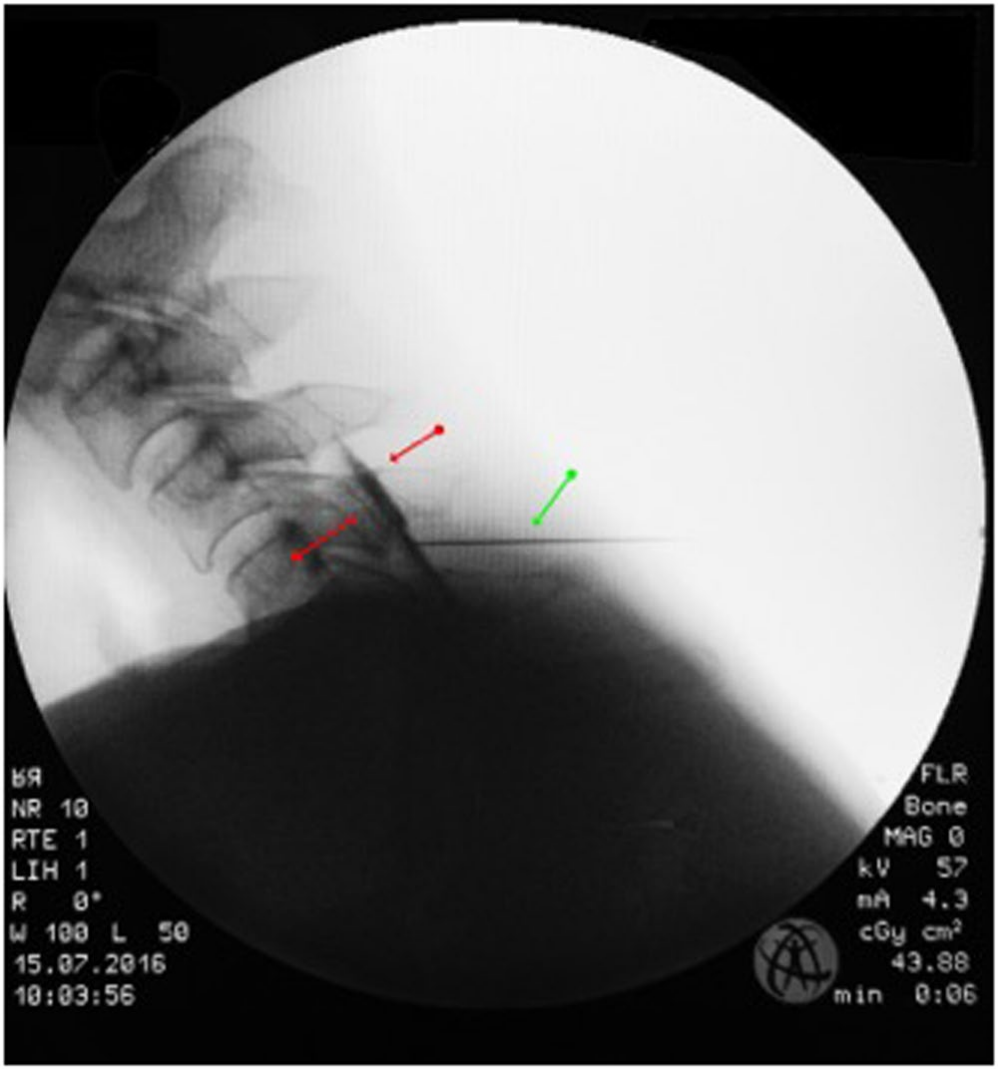
Typical pattern of epidural contrast agent distribution between red arrows; green arrow: needle.

On average, each patient received two injections daily, one in the morning and one at noon. The injections contained 0.5% Mepivacaine, a cervical spinal nerve root analgesic (CSPA), which was injected into the affected nerve root in ‘free-hand technique’^21, 43^. Additional treatment consisted of 1 cervical epidural translaminar injection per hospital stay conducted in ‘loss-of-resistance technique’ under X-ray control in 2 planes in the operating theatre^21, 43, 44^. Cervical epidural translaminar injections contained 40 mg of triamcinolone and the contrast agent solutrast 250 (Fig. 2). The other applications have already been described by the author^18, 32^: ‘Physiotherapy and sports therapy as part of inpatient MPM includes group exercises and aqua training; accompanying measures consist of electrotherapy for muscle relaxation and thermotherapy. In addition, patients are instructed in progressive muscle relaxation according to Jacobsen and take part in coordination training^45^. The most effective exercises are isometric exercises for strengthening the neck muscles, which is further aided by medical training therapy with workout equipment. The main goal is recovery of the load-bearing capacity and reduction in pain-avoidance behaviour. To address pain management, patients participate in psychological trainings and interviews with psychologists. The success of MPM depends on accurate patient information and consultation, continuous motivation, a systematic increase of load, and permanent feedback’. Each patient had to keep a pain diary to note the pain score 4 times a day. The average schedule of a 10-day program is shown in Table 2.

**Table 2:** Example of a 10-day schedule of physiotherapy and sports therapy as part of inpatient MPM.

Exercise	Number
Group exercises	4
Aqua training	5
Neck exercises	5
Instructions on progressive muscle relaxation	3
Psychological behavioral education	3
Coordination training group	4

### Data

Data were recorded daily in a standardized manner. The data obtained before, during, and after treatment were compared to assess the treatment success at the end of hospitalization. Besides the numerical rating scale (NRS) for neck and arm pain as a main evaluation criterion, the validated German version of the Neck Disability Index (NDI)^46^ was assessed at the beginning and at the end of therapy. Minimal clinically significant pain reduction was set to NRS 2.0^47–49^. Treatment success was defined as NRS < 50% of the initial pain at the end of hospitalization^50^. Using the NDI, a score of < = 8% means patients with hardly any symptoms, whereas a score >40 stands for patients with severe problems and a higher risk of pain chronification^51–55^. The minimal clinically relevant change has ranged from 3.5% to 9.5% depending on the respective author^53, 54^. We used the percentage of 8.4% applied by Jorritsma *et al*.^55^. The Hospital Anxiety and Depression Scale-German version (HADS-D) were evaluated only on the first day of therapy, because no changes were expected during hospitalization. Scores from 0 to 7 are normal, scores from 8 to 10 borderline abnormal, and scores higher than 10 are abnormal for both anxiety and depression^56, 57^.

To assess the effect of cervical epidural injection as conducted for triamcinolone without any local anaesthetics, pain reduction was evaluated 2 days after injection^58^. Because these injections were only given on Tuesdays or Fridays, some sort of randomisation was provided on what day of hospitalisation the epidural injection was given. Patients were admitted to hospital from Monday to Friday. At the end of the stay all collected data were pseudonymized saved. Primary outcome was pain reduction >NRS 2 for arm and neck pain.

### Statistical analysis

Statistical analysis was done with SPSS (IBM SPSS Statistics, Version 23.0., Armonk, NY: IBM Corp.). Metric variables were reported descriptively as mean and standard deviation. Statistical data were not normally distributed. Data were compared with the non-parametric Wilcoxon test. The level of significance was set at p < 0.05. A sample size of n = 54 resulted in 80% power to detect a significant effect, if the true effect size of the total population was d = 0.39, which can be considered small.

The datasets generated during and/or analyzed during the current study are available from the corresponding author on reasonable request.

## Results

### Questionnaires

The Neck Disability Index (NDI) is used to measure everyday impairment. The mean value at the initial examination on day 1 was 37.1%. As described above, the minimal clinically relevant change was set at 8.4%. There was a significant improvement (p < 0.001), 57.4% of the patients described pain reduction of more than 8.4%. The mean changes were 11.24% but started at −8, which means pain increase in one patient and pain reduction in 52% of patients.

The Hospital Anxiety and Depression Scale (HADS-D) was additionally used to detect possible psychological abnormalities^56^. The results for anxiety and depression can be seen in Table 1. During the first day assessment, patients were also asked about existing comorbidities. 10 patients (18.5%) stated depression, and the HADS showed 20% abnormalities on the depression scale. Interestingly, only 5 of these 10 patients also had an abnormal HADS score.

### Pain

**Figure 3:**
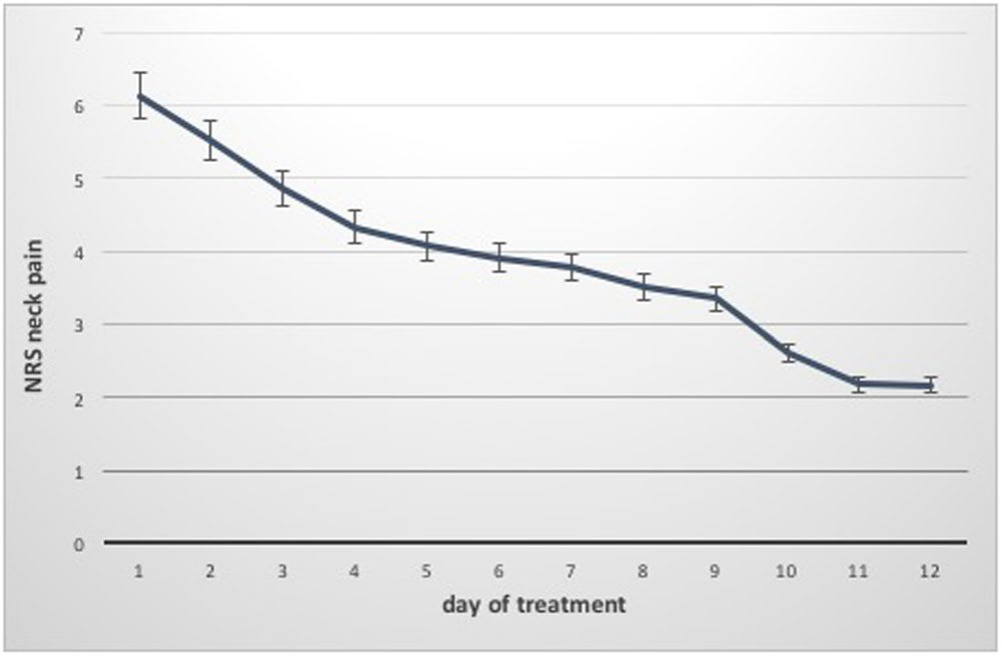
Course of neck pain during hospitalization (Median and IQR).

**Figure 4:**
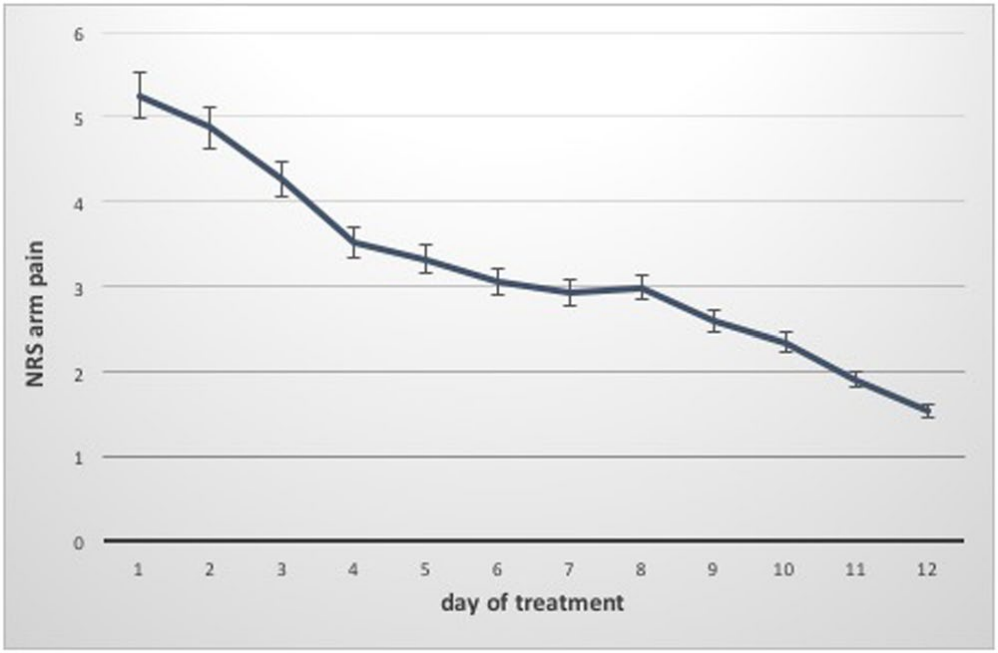
Course of arm pain during hospitalization (Median and IQR).

The median NRS value for neck pain was 6.0 (5.7–6.8) before treatment and 2.25 (2.0–3.1) at discharge. The NRS value for arm pain could be reduced from 5.9 (4.8–6.0) to 2.0 (1.7–2.6). (Table 3, Fig. 3 and 4) The median pain reduction at the end of treatment was 3.5 (3.1–4.1) for neck pain and 3.0 (2.7–3.8) for arm pain, which means a reduction in neck pain by 57.4% and a reduction in arm pain by 62.5%. Both reductions were statistically significant (p < 0.05). Pain reduction in women was higher than that in men, but this difference was not significant. As described above, the minimal clinically relevant reduction in NRS was set at 2. This value was reached after 4.0 (3.8–5.2) days for neck pain and after 4.0 (3.4–4.8) days for arm pain. According to the IMMPACT definition of treatment success with an NRS improvement of 50% at the end of hospitalization, 40 patients (74.1%) were treated successfully for neck pain and 36 (66.7%) for arm pain^50^.

**Table 3:** Data on pain treatment of the patient group (median and interquartile range; mean and standard deviation).

	Women (n = 32)	Men (n = 22)	Together (n = 54)
Treatment days	10.0 (9.3–10.7) 10 (±1.7)	10.0 (9.4–11.0) 10.2 (±1.7)	10.0 (9.6–10.6) 10.1 (±1.7)
Neck pain on day 1 (NRS)	6.0 (5.3–7.0) 6.0 (±1.9)	6.75 (6.7–7.2) 6.4 (±1.6)	6.0 (5.7–6.8) 6.1 (±1.8)
Arm pain on day 1 (NRS)	5.75 (4.5–6.3) 5.5 (±2.1)	6.0 (4.6–6.4) 5.5 (±1.9)	5.9 (4.8–6.0) 5.5 (±2.0)
Neck pain on day of discharge (NRS)	2.25 (1.7–3.5) 2.7 (±2.1) P = 0.000	2.25 (1.8–3.1) 2.4 (±1.4) P = 0.000	2.25 (2.0–3.1) 2.6 (±1.9) P = 0.000
Arm pain on day of discharge (NRS)	1.75 (1.4–2.6) 2.0 (±1.5) P = 0.000	2.0 (1.5–3.1) 2.3 (±1.7) P = 0.000	2.0 (1.7–2.6) 2.1 (±1.6) P = 0.000
Days of hospitalization needed for relief of neck pain >2 (NRS)	4.0 (3.0–5.3) 4.1 (±2.0)	5.0 (4.1–5.6) 5 (±1.8)	4.0 (3.8–5.2) 4.4 (±1.9)
Days of hospitalization needed for relief of arm pain >2 (NRS)	4.0 (2.8–4.8) 3.7 (±2.3)	4.0 (3.5–5.5) 4.5 (±2.3)	4.0 (3.4–4.8) 4.1 (±2.3)

### Cervical translaminar epidural steroid injection

In the mean, cervical translaminar epidural steroid injection was given on day 4, but this time point ranged between day 1 and 9. 2 days after epidural steroid injection, pain was reduced by 40.1% in the neck and by 43.4% in the arms. Adverse events were noted in the diary by the patients. Only 4 (7.4%) patients described some dizziness as a side effect, but no severe adverse events were reported.

## Discussion

The study aimed at showing the positive short-term effect of an inpatient multimodal therapeutic concept based on drug injections for patients with cervical radiculopathy with particular focus on cervical translaminar epidural steroid injection.

Within this concept, we showed that neck pain improved from NRS 6.0 to 2.25 and arm pain from 5.9 to 2.0, and both values represent significant pain reduction (p < 0.001).

68.5% (37) of the treated patients had experienced pain for more than 3 months. Thus, avoiding pain chronification or trying to reverse the effects is all the more important. The NDI score as a value for every day impairment had also improved during hospitalization.

Only single methods are usually described as treatment options. Most studies have either compared different surgical approaches^59^ or the type of injection^10, 24–28, 60–65^. Conservative treatment options have been recommended in many reviews on cervical radiculopathy, ranging from different types of physiotherapy to waiting for remission by natural history. However, no multimodal treatment concept on an inpatient basis has been evaluated so far^9, 39, 40^.

In times of low frequency outpatient treatment, first pain relief takes longer, bearing a high risk of pain chronification. In this study, the period of the first clinically relevant success with regard to pain reduction of more than NRS 2 was 4.4 days for neck pain and 4.1 days for arm pain. Because of the combination of several types of conservative treatment and 2 injections per day, patients receive different conservative treatments over a very short period of time, which leads to quick pain relief and thus − in combination with psychological lessons – tries to avoid chronification^32^.

Particularly physical activation is the key to successful conservative treatment. ‘The aim of the accompanying methods is to preserve or restore the full capacity of the spine-stabilizing muscles, to train coordination skills and to learn spine friendly behaviour. Risk factors that can lead to chronic pain must be eliminated. All methods serve to interrupt the vicious circle of “pain−stress−malposition−pain”^32^. While reducing pain through injections, patients may take part in activating treatments as mentioned above. ‘Besides direct physical effects, the accompanying therapies also help to improve the affective-emotional level as well as the motor-psychomotor level by learning physically regulating movements. The focus is on activating methods, not on passive methods’^32^.

With this study, we showed that there is the wanted effect on neck and arm pain. Therefore, these data also support our concept of interrupting the vicious circle of ‘pain–stress–malposition–pain’. It is clear, that a restauration of the full capacity of the spine-stabilizing muscles cannot be achieved after just 10 days, but this intensive treatment should be the cornerstone for further exercises.

Many studies show positive effects of different types of exercises. Cervical range of motion and strengthening exercises are typically not recommended when patients are symptomatic as these movements may exacerbate symptoms^66^. This supports our idea of reducing pain by injections first. Cervical traction and immobilization has been supported in some studies to calm the symptoms associated with cervical radiculopathy^67^. Also cervical traction may increase the dimensions of the neuroforamen by distraction. A recent prospective randomized clinical trial found that adding mechanical traction to an exercise protocol in patients with cervical radiculopathy resulted in superior outcomes (neck disability index, arm pain) when compared with exercise plus over door traction, or exercise alone^68^. As traction was not included in our therapy yet, further investigation of this effect within our concept will be interesting. It has also been shown that neck-specific exercises with and without behavioral approach has better results than general physical activity^36^.

Another important part of the MPM are the psychological lessons. Psychosocial interventions should be offered in addition to the medical treatment to patients to reduce the risk of chronic pain. The efficacy of such an interdisciplinary care has been shown^69^.

In this study, special focus was placed on the effect of cervical translaminar epidural steroid injection. Although, epidural injections have now been used for many years and are the only effective, non-operative tool for managing cervical radiculopathy, the use of corticosteroid is still ‘off-label’ worldwide^22^. In addition, many reports have described severe complications, but Pountos *et al*. stated in their review that ‘the true incidence of such complications remains unclear.’ The most devastating complications such as paralysis are very rare and have been limited to case reports^64^. Minor adverse events have an incidence of less than 1% and are generally transient^42^. But controversial discussions are still part of the scientific literature. There even was a Food and Drug Administration (FDA) warning on April 23, 2014, with regard to injecting corticosteroids into the epidural space of the spine^70, 71^. Other reviews have described cervical epidural steroid injections as ‘effective in easing pain and reducing the need for surgery’, although ‘the evidence of effectiveness is of very low quality, and the benefits of the procedure are compromised by the risks of serious complications’^23, 58, 61^. Furthermore, the use of non‐particulate vs. particulate steroids has been discussed. In our opinion, particulate steroids, similar to triamcinolone, remain locally effective far longer because the particles remain at the site of the injection^21, 72^. On the other hand, this mechanism may lead to the described severe complications because of intravascular injection^73^. In addition, Shakir *et al*. showed that transforaminal epidural steroid injections have similar benefits, independent of the type of corticosteroid formulation used (triamcinolone or dexamethasone)^58^. After analysis of the complications, the FDA recommended an interlaminar approach instead of a transforaminal approach as well as the use of non-particulate steroids to minimize the risk of accidental arterial uptake and neurological damage^71^. In our therapy concept, we always use the interlaminar approach as described by Grifka *et al*.^21^. Over the past 16 years, we have always used triamcinolone as particulate steroid without any severe complications. In our study population, 4 patients described some dizziness for a few hours after the injection. Because these injections are given in our operating theatre, we often observe this phenomenon that is mainly caused by higher blood pressure due to the agitation of the patients. Thus, in our multimodal pain concept, epidural steroid injection has been a very effective tool for reducing pain in addition to the ‘normal’ daily injections.

The biggest limitation of this study may be the lack of a general control group. However, implementation of a control group is difficult when evaluating the overall concept of MPM and not just the sub-item ‘injections’. Moreover, study populations may have some selection bias because patients are only included after failed outpatient unimodal therapy. Therefore, study populations may have a trend towards chronification. Another possible bias might be, that the data collection was not anonymous and therefore the patients might want to please their caregiver. Another limitation is the short follow-up and the effect after discharge is not known. Future studies will have to show the mid- and long-term effect of this therapy.

## Conclusion

In summary, this study showed MPM based on injections to be an efficient treatment option for cervical radiculopathy. Despite several reports on severe complications published in the literature, MPM appears to be a safe procedure, and transforaminal epidural steroid injection may be an important factor of this concept. In the absence of an absolute indication for surgery, this is a treatment option that could be tried before surgery.

### Ethical approval and informed consent

The study was approved by the Ethics Commission of the University of Regensburg and carried out in accordance with the approved guidelines. Registration in Deutsche Register Klinischer Studien (DRKS), German Clinical Trials Register DRKS00011788.
